# Combination of Neovestitol and Vestitol Modifies the Profile of Periodontitis-Related Subgingival Multispecies Biofilm

**DOI:** 10.3390/biomedicines12061189

**Published:** 2024-05-27

**Authors:** Tatiane Tiemi Macedo, Larissa Matias Malavazi, Gustavo Quilles Vargas, Francisco Jerfeson dos Santos Gonçalves, Aline Paim de Abreu Paulo Gomes, Manuela Rocha Bueno, Lucas Daylor Aguiar da Silva, Luciene Cristina Figueiredo, Bruno Bueno-Silva

**Affiliations:** 1 Dental Research Division, Guarulhos University, Guarulhos 07023-070, SP, Brazil; tatianet92@gmail.com (T.T.M.); francisco.jerfeson@outlook.com (F.J.d.S.G.); daylor.estudos@gmail.com (L.D.A.d.S.); lucienedefigueiredo@gmail.com (L.C.F.); 2 Departamento de Biociências, Faculdade de Odontologia de Piracicaba, Universidade de Campinas (UNICAMP), Piracicaba 13414-903, SP, Brazil; l272371@dac.unicamp.br (L.M.M.); g272372@dac.unicamp.br (G.Q.V.); 3 Faculdade São Leopoldo Mandic, Campinas 13045-775, SP, Brazil; manuela.bueno@slmandic.edu.br

**Keywords:** periodontitis, red propolis, antimicrobial, oral microorganism

## Abstract

The aim of this study was to evaluate the effect of the combination of neovestitol–vestitol (CNV) compounds obtained from Brazilian red propolis on the microbiological profile of a mature multispecies subgingival biofilm. The biofilm with 32 bacterial species associated with periodontitis was formed for seven days using a Calgary device. Treatment with CNV (1600, 800, 400, and 200 μg/mL), amoxicillin (54 μg/mL), and vehicle control was performed for 24 h on the last day of biofilm formation. Biofilm metabolic activity and DNA–DNA hybridization (checkerboard) assays were performed. The groups treated with CNV 1600 and amoxicillin reduced 25 and 13 species, respectively, compared to the control vehicle treatment (*p* ≤ 0.05); both reduced *P. gingivalis*, while only CNV reduced *T. forsythia*. When the data from the two treatments (CNV and AMOXI) were compared, a statistically significant difference was observed in 13 species, particularly members of Socransky’s orange complex. Our results showed that CNV at 1600 μg/mL showed the best results regarding the metabolic activity of mature biofilms and obtained a reduction in species associated with the disease, such as *T. forsythia*, showing a better reduction than amoxicillin. Therefore, CNV seems to be a promising alternative to eradicate biofilms and reduce their pathogenicity.

## 1. Introduction

Periodontal disease represents a pathology of significant importance for oral health; its development is intrinsically linked to a complex pathogenic dental biofilm, which shelters multiple microbial communities capable of evading the defenses of the immune system [1]. As a result, an uncontrolled immuno-inflammatory response starts in the host, generating progressive damage to the protective and supporting periodontium [2]. When these microbial communities are in balance with the defense system, they have a higher proportion of bacteria associated with health and the periodontal tissues maintain health; however, oral biofilm dysbiosis associated with an exacerbated immuno-inflammatory response in the host is evidenced by the development of periodontal pockets and tooth mobility [3].

Numerous preventive strategies have been employed to reduce the number of microorganisms and, in consequence, decrease inflammation response and thus reduce the risk of periodontal disease [4]. The mechanical removal of biofilm (scaling and root planning) is considered the conventional treatment, although it is not always sufficient. Consequently, in many cases, it is essential to consider the use of adjunctive treatments, for example, systemic antibiotics like the combination of amoxicillin and metronidazole [5] or probiotics [6].

Although antibiotic therapy has been shown to improve the clinical parameters of periodontal disease, its effects on the human body can cause changes in the composition of the microbiota of other body systems due to its broad spectrum and prolonged use [7,8,9], as well as the risk of bacterial resistance, so the discovery of new drugs from natural products has aroused considerable interest in the scientific community.

Brazilian propolis is a natural substance that has a variety of pharmacological properties, including antimicrobial, antioxidant, anticancer, and anti-inflammatory activities [10,11,12]. The composition of Brazilian propolis is highly variable due to the presence of multiple components, resulting in a wide chemical diversity of bioactive compounds that vary according to the geographical location of its production [10,11].

Brazilian red propolis (BRP) is produced by *Appis mellifera* bees and originates mainly in the coastal region of Maceió city, Alagoas state. Its botanical origin is the resin of *Dalbergia ecastophyllum* [13]. This propolis has an intense red color and its main constituents are isoflavonoids and flavonoids, especially formononetin, vestitol, and neovestitol [10]. Neovestitol and vestitol appear to be a great option as local oral bioactive agents due to their antimicrobial and anti-inflammatory properties [14,15,16,17].

The natural combination of neovestitol–vestitol (60/30%) showed a minimum inhibitory concentration of 50 µg/mL against *Porphyromona gingivalis* and *Aggregatibacter actinomycetemcomitans*, inhibited the formation and development of *Streptococcus mutans* biofilm in vitro [18], and reduced the development of dental caries through an in vivo study at a concentration of 800 µg/mL [19], demonstrating its potential to inhibit the growth of microorganisms important in the development of biofilm associated with periodontal disease. Therefore, the aim of this study was to evaluate the effect of the combination of neovestitol–vestitol (CNV) compounds obtained from Brazilian red propolis on the microbiological profile of mature multispecies subgingival biofilm.

## 2. Materials and Methods

### 2.1. Biofilm Formation

The bacterial species below were used in the multispecies biofilm model: *Actinomyces naeslundii* ATCC12104, *Actinomyces oris* ATCC43146, *Actinomyces gerencseriae* ATCC23840, *Actinomyces israelii* ATCC12102, *Veillonella parvula* ATCC10790 *Actinomyces odontolyticus* ATCC17929, *Streptococcus sanguinis* ATCC10556, *Streptococcus oralis* ATCC35037, *Streptococcus intermedius* ATCC27335, *Streptococcus gordonii* ATCC10558, *Streptococcus mitis* ATCC49456, *Aggregatibacter actinomycetemcomitans* ATCC29523, *Capnocytophaga ochracea* ATCC33596, *Capnocytophaga gingivalis* ATCC33624, *Eikenella corrodens* ATCC23834, *Capnocytophaga sputigena* ATCC33612, *Streptococcus constellatus* ATCC27823, *Eubacterium nodatum* ATCC33099, *Fusobacterium nucleatum vincentii* ATCC49256, *Parvimonas micra* ATCC33270, *Fusobacterium nucleatum polymorphum* ATCC10953, *Campylobacter showae* ATCC51146, *Fusobacterium periodonticum* ATCC33693, *Prevotella intermedia* ATCC25611, *Porphyromonas gingivalis* ATCC33277, *Tannerella forsythia* ATCC43037, *Eubacterium saburreum* ATCC33271, *Streptococcus anginosus* ATCC33397, *Selenomonas noxia* ATCC43541, *Propionibacterium acnes* ATCC11827, *Gemella morbillorum* ATCC27824, and *Streptococcus mutans* ATCC25175.

The microorganisms were grown on tryptone soy agar with 5% sheep’s blood under anaerobic conditions (85% nitrogen, 10% carbon dioxide, and 5% hydrogen), with *P. gingivalis* and *P. intermedia* being grown on yeast extract enriched with 1% hemin, 5% menadione, and 5% sheep’s blood, while *T. forsythia* was grown on tryptone soy agar with yeast extract enriched with 1% hemin, 5% menadione, 5% sheep’s blood, and 1% N-acetylmuramic acid. After 48 h of growth, all bacterial species were transferred to BHI broth (Becton Dickinson, Sparks, MD, USA) supplemented with 1% hemin.

After 24 h, the optical density (OD) at 600 nm was adjusted to 0.1, corresponding to around 10^8^ cells/mL of each species. The individual cell suspensions were diluted to obtain the final biofilm inoculum, containing 10^4^ cells of each species. The multiple species model of subgingival biofilm was developed using the Calgary biofilm device (CBD) according to Soares et al. [20].

### 2.2. CNV Obtainment

CNV was obtained naturally through the fractionation of Brazilian red propolis (BRP), as described previously [19]. The red propolis studies were registered in the National System for the Management of Genetic Heritage and Associated Traditional Knowledge of the Brazilian Federal Government (SISGEN—register number A305815). Briefly, BRP samples were collected in the State of Alagoas, Brazil, and first an ethanolic extract was prepared. Liquid–liquid fractionation with two solvents (hexane and chloroform) was then performed on the ethanolic crude BRP extract. As previously described [18], the chloroform fraction has better antimicrobial activity and was subjected to two different chromatographic separations: dry column (polarity separation) and Sephadex LH-20 column (size separation). The combination of Neovestitol and Vestitol compounds used here was obtained after Sephadex LH-20, and a previous work characterized its chemical composition and demonstrated its reducing effect on *S. mutans* biofilm [19].

### 2.3. 24 h Treatment with CNV on Mature Biofilm

The biofilms were formed for 7 days, and the medium was changed on day 3 (after 72 h of incubation) and day 6 (after 144 h of formation). After 6 days of formation, the biofilms were treated for 24 h until the completion of 7 days of formation. For the treatment, the pins coated with the biofilms were washed twice with 200 µL of 1% PBS and transferred to 96-well plates containing culture medium (100 µL) and 100 µL of different concentrations of CNV-VO (1600; 800; 400 and 200 µg/mL), chosen according to previous results with crude propolis [21,22]. For this therapeutic scheme, in addition to the untreated control group, a group treated with amoxicillin at 54 µg/mL was also included (concentration chosen according to Soares et al. [20]). At the end of seven days of biofilm formation, items 2.4. (metabolic activity) and 2.5. (checkerboard) [20], described below, were carried out.

### 2.4. Biofilm Metabolic Activity

The percentage reduction in biofilm metabolic activity was determined using 2,3,5-triphenyltetrazolium chloride (TTC) and spectrophotometry. TTC is used to differentiate between metabolically active and inactive cells. The white substrate was enzymatically reduced to red 1,3,5-triphenyl formazan (TPHP) by living bacterial cells due to the activity of various dehydrogenases enzymes. The substrate color change was read using spectrophotometry to determine the rate of reduction, which is used as an indirect measure of bacterial metabolic activity. To measure the metabolic activity of the biofilms, the pins were washed twice with washing solution and transferred to plates with 200 µL per well of fresh BHI medium containing 1% hemin and 0.1% TTC solution. The plates were then incubated under anaerobic conditions for 6–8 h at 37 °C. TTC conversion was read at 485 nm using a spectrophotometer [20].

### 2.5. DNA–DNA Hybridization (Checkerboard)

Calgary device “pegs” coated with 7-day biofilm from each of the groups were washed twice with PBS and transferred to Eppendorf tubes containing 150 µL of TE buffer (Tris 10 mM -HCl, EDTA 1 mM [pH 7.6]), and then 100 µL of 0.5 M NaOH was added. The tubes containing the pins and the final solution were boiled for 10 min, and the solution was neutralized by adding 0.8 mL of 5 M ammonium acetate. The samples were then analyzed individually for the presence and quantity of the 30 bacterial species using the DNA–DNA hybridization technique. Briefly, after the lysis of the samples, the DNA was placed in lanes on a nylon membrane using a Minislot device (Immunetics, Cambridge, MA, USA). After fixing the DNA to the membrane, which was placed in a Miniblotter 45 (Immunetics), digoxigenin-labeled whole-genome DNA probes for the subgingival species used were hybridized to individual lanes of the Miniblotter 45. After hybridization, the membranes were washed, and the DNA probes were detected using an antibody specific for digoxigenin conjugated with alkaline phosphatase. The signals were detected using AttoPhos substrate (Amersham Life Sciences, Arlington Heights, IL, USA), and the results were read using Typhoon Trio Plus (Molecular Dynamics, Sunnyvale, CA, USA). Two lanes in each run contained the standards with 10^5^ or 10^6^ cells of each species. Signals obtained with the Typhoon Trio were converted into absolute counts by comparison with the standards on the same membrane. Failure to detect a signal was recorded as zero. The values obtained after treatment with the samples were compared with the pegs of the positive and negative control groups.

### 2.6. Statistical Analysis

Data from the biofilm’s metabolic activity test were statistically analyzed using Analysis of Variance (ANOVA) followed by Tukey’s test. The results of the checkerboard DNA–DNA hybridization were statistically analyzed using Kruskal–Wallis, followed by Dunn’s post hoc (*p* ≤ 0.05).

## 3. Results

**Figure 1:**
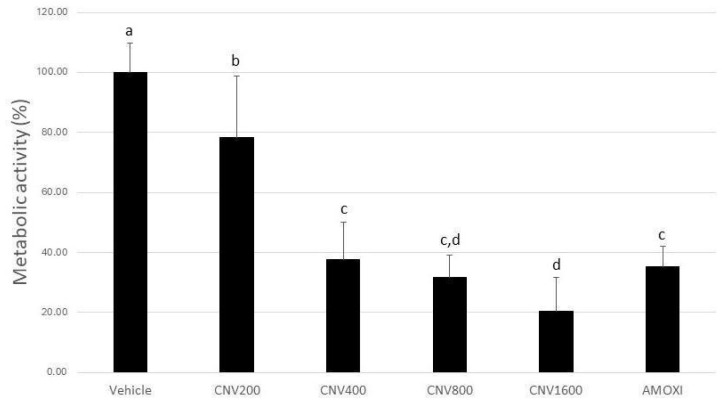
Effect of CNV treatment at 200, 400, 800, and 1600 µg/mL and amoxicillin at 54 µg/mL for 24 h on the metabolic activity of biofilms formed over 7 days (n = 10–3 separate experiments). Different letters mean statistically significant differences among the groups determined using Kruskal–Wallis’s test, followed by Dunn’s post hoc test (*p* ≤ 0.05).

Biofilms treated with CNV at 1600, 800, 400, and 200 µg/mL, and amoxicillin showed a statistically significant reduction in metabolic activity of 79, 68, 62, 21, and 65%, respectively when compared to the negative control group (*p* < 0.05) (Figure 1). The metabolic activity of mature biofilms treated with CNV at 1600 µg/mL showed the lowest absolute value (21%), showing better results even than amoxicillin (*p* < 0.05). The group treated with 800 µg/mL showed no statistically significant difference to the CNV 400, CNV 1600, and AMOXI groups (*p* > 0.05) (Figure 1).

**Figure 2:**
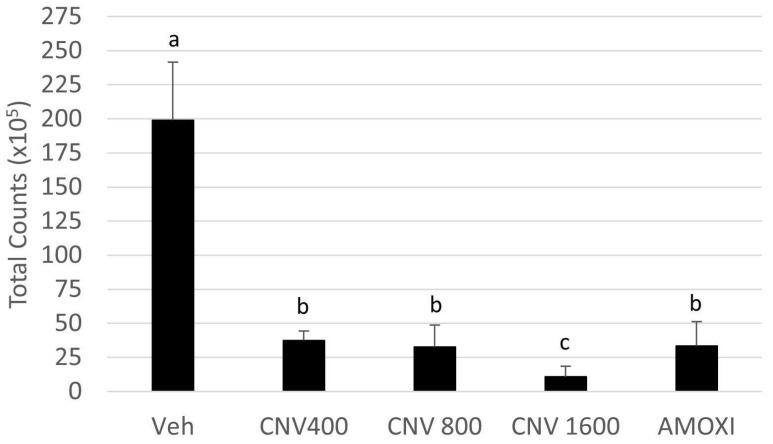
Mean and standard deviation of the total count (×10^5^) of biofilms formed over 6 days and treated for 24 h with control vehicle (Veh), CNV at 400, 800, and 1600 µg/mL and amoxicillin at 54 µg/mL (n = 9–3 separate experiments). Different letters mean statistically significant differences among the groups determined using Kruskal–Wallis’s test, followed by Dunn’s post hoc test (*p* ≤ 0.05).

Figure 2 shows the results of the total biofilm count formed over 7 days and treated with CNV at 1600, 800, and 400 µg/mL, amoxicillin, and control vehicle. As the group treated with CNV at 200 µg/mL showed the worst metabolic activity results, it was discarded from subsequent analyses. The group treated with CNV at 1600 µg/mL showed the lowest total count values, corroborating the metabolic activity data. The groups treated with CNV at 400 and 800 µg/mL showed no statistically significant difference to the group treated with amoxicillin (positive control).

**Figure 3:**
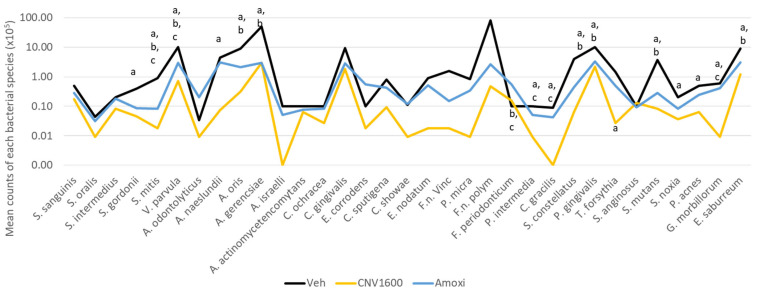
Average counts of each of the bacterial species present in the biofilm. Statistical analysis carried out using Kruskal–Wallis’s test, followed by Dunn’s post hoc test (*p* ≤ 0.05). The letter “a” means statistical difference between CNV1600 and Vehicle; the letter “b” means statistical difference between Vehicle and Amoxicillin (AMOXI); and the letter “c” means statistical difference between CNV1600 and AMOXI.

Figure 3 shows the average count results for each species used in the biofilm model treated with Vehicle, CNV 1600, and AMOXI. Since the 1600 µg/mL concentration of CNV showed the most promising results in the previous tests, only this concentration was selected for the analysis of the count of each species. The treatments with CNV 1600 and AMOXI reduced 25 and 13 species, respectively, compared to the control vehicle treatment (*p* ≤ 0.05). It is noteworthy that both treatments reduced *P. gingivalis* counts, while only CNV reduced *T. forsythia* values. When comparing the data from both treatments (CNV and AMOXI), a statistically significant difference was observed in 13 species, highlighting *P. intermedia*, *F. periodonticum*, *F. nucleatum polymorphum*, *F. nucleatum vicentii*, and *P. micra* (all members of Socransky’s orange complex).

## 4. Discussion

The present article shows the effects of a combination of Neovestitol and Vestitol, a fraction isolated from Brazilian red propolis, on the periodontitis-related subgingival multispecies biofilm. Three different concentrations of CNV (1600, 800, and 400 µg/mL) were shown to have a reducing effect on the mature biofilm. The highest tested concentration (1600 ug/mL) was even better than the amoxicillin treatment, reducing the amount of 25 species compared to vehicle control and 13 species compared to amoxicillin-treated biofilms. It seems that amoxicillin is a non-selective antibiotic, acting on both health- and disease-associated bacteria, while CNV has a prominent effect on disease-associated species.

The dysbiotic subgingival biofilm is an important etiological factor of periodontitis. It is a complex, intricate community formed by several distinct bacterial species and other microorganisms. Initially, the bacteria found in periodontitis patients were divided into five groups, according to their association with healthy or diseased conditions [23]. The main species associated with diseased sites were identified as *P. gingivalis*, *T. forsythia*, and *Treponema denticola*. Nowadays, researchers are searching for novel putative oral pathobionts [24,25]. In recent studies, other species, such as *Filifactor allocis*, have been investigated as putative new pathogens, and a clinical investigation confirmed their presence in patients with periodontitis [26]. These results suggest that the composition of the biofilm associated with periodontal disease may be more complex and diverse than previously thought [27,28]. In this context, it has been shown that a consortium of bacteria, rather than a single species, is accountable for the transition of the biofilm from a state associated with oral health to one that is pathogenic. Notably, *P. gingivalis*, along with *T. forsythia* and *S. gordonii* (and potentially other oral bacteria), have been identified as significant contributors to the dysbiosis of the subgingival biofilm, which ultimately results in the development of periodontal disease [29].

*P. gingivalis* may be considered the primary periodontal pathogen responsible for biofilm dysbiosis since it has been identified as a keystone pathogen in periodontal disease due to its production of various virulence factors (for example, gingipains and FimA), with properties to subvert the human immune response, including neutrophils, macrophages, and the complement system [30]. Therefore, the effect of CNV on *P. gingivalis* is remarkable. The natural compound combination reduced the quantity of this keystone pathogen in the mature biofilm by almost fivefold.

Of particular note, only the biofilms treated with CNV at 1600 µg/mL exhibited a statistically significant reduction in *T. forsythia* (Figure 3), another periodontal pathogen related to biofilm dysbiosis. Even amoxicillin was not statistically significant in reducing the amounts of this pathogen when compared to vehicle-treated biofilms. A recent article described that, during the infection of macrophages by *T. forsythia*, the immune response was led to produce pro-inflammatory factors (such as BspA, sialidase, GroEL, and numerous lipoproteins) related to periodontal tissue damage through the activation of the Toll-like 2 receptor of macrophages [31].

Another significant observation is the decline in all species of the *Fusobacterium* genera included in the model (*F. nucleatum vincentii*, *F. nucleatum polymorphum*, and *F. periodonticum*) by CNV at a concentration of 1600 µg/mL. The *Fusobacterium* genera are notably influential in the transition from periodontal health to disease [32]. *F. nucleatum* is recognized as the most prevalent anaerobic, Gram-negative species during the advanced stages of periodontal disease, and has been suggested as a potential periodontal pathogen [33]. According to research by Colombo et al. (2002) [34] and Socransky and Haffajee (2002) [33], the presence of *F. nucleatum* is predominantly associated with individuals affected by periodontitis and periodontal abscesses. Furthermore, its abundance tends to decrease after successful periodontal therapy [32]. Acting as an intermediary colonizer within dental biofilms, *Fusobacterium* species are among the first Gram-negative organisms to establish a stable presence in the subgingival biofilm. This pivotal role involves facilitating interactions between Gram-positive and Gram-negative species, thereby fostering the colonization of additional anaerobic species, including those recognized as pathogens within the red complex [32].

The present in vitro biofilm model is widely used by our research group. The number of bacterial species included in the model is the main strength since, to our knowledge, this is the only model that includes almost all Socransky species (except *T. denticola*). The values for the reduction in metabolic activity of biofilms treated with amoxicillin are consistent with those obtained in previous studies [20,22,35], demonstrating the reproducibility of the method. It is of importance to note that among all the articles published to date with this therapeutic regimen (treatment for 12 or 24 h), CNV at 1600 µg/mL was the first test agent to show metabolic activity and total counts significantly lower than the positive control, either amoxicillin or chlorhexidine [22,35,36,37]. This result is considered very promising. Another point to note is that as this formulation is intended to be used orally, some metabolization of the bioactive compounds is to be expected. Although not carried out with the same propolis or CNV compounds, a recent study evaluated the in vitro metabolization of organic propolis containing flavonoid compounds. The results showed that the concentration of flavonoid compounds in propolis was reduced by about ¼ after gastric and intestinal metabolization [38]. It is therefore important to note that CNV at 400 µg/mL showed statistically similar results to the biofilm treated with the positive control amoxicillin, demonstrating that the oral intake of 1600 µg/mL may be effective through in vivo studies; however, this hypothesis needs to be tested in future works.

Furthermore, the efficacy of systemic antibiotics in treating periodontal disease is well documented in the literature [39]. However, it is important to acknowledge the potential side effects associated with prolonged antibiotic therapy (lasting 7 or 14 days), which include disrupting the resident microbiota, the selection of antimicrobial-resistant organisms [7,40], damage to the intestinal microbiome [41], and even microbial alterations in the skin [42]. The toxicity of the compounds Neovestitol and Vestitol is another issue that needs to be investigated. To date, in vivo studies in animals (mice and rats) have not reported any signs of toxicity after the use of the compounds [19,43,44], but future studies should focus specifically on toxicity. Another matter to be investigated in the future would be the possible effects on the gut microbiome. These findings underscore the potential value of conducting future in vivo studies on CNV to explore its potential benefits in treating periodontitis.

## 5. Conclusions

In conclusion, the combination of the compounds neovestitol and vestitol (CNV) proved to be effective in disrupting the mature multispecies subgingival biofilm associated with periodontal disease. The results indicate that CNV concentrations of 800 and 400 µg/mL reduced the levels of classical periodontal pathogens in a manner similar to amoxicillin, while CNV at 1600 µg/mL showed the best results with respect to the metabolic activity of mature biofilms and achieved a better reduction in disease-associated species such as *T. forsythia* than the positive control amoxicillin. Therefore, CNV appears to be a promising alternative to eradicate biofilm and decrease its pathogenicity. However, further studies are needed to corroborate and extend these results.

## Data Availability

Data will be available upon request.
